# Evaluation of protein production in rice seedlings under dark conditions

**DOI:** 10.1038/s41598-022-11672-0

**Published:** 2022-05-11

**Authors:** Akiko Watanabe, Yoshino Hatanaka, Yukino Takeshima, Karin Sasaki, Noa Takahashi, Yukihiro Ito

**Affiliations:** 1 Graduate School of Agricultural Science, Tohoku University, Sendai, 980-8572 Japan; 2 Exploring-Germination-and-Growth Program for Young Scientists, Tohoku University, Sendai, 980-8579 Japan; 3 Takasaki Girls High School, Takasaki, 370-0062 Japan; 4 Akita High School, Akita, 010-0851 Japan; 5 Renaissance High School, Daigo-machi, Ibaraki, 319-3702 Japan; 6 Sakata Higashi High School, Sakata, 998-0842 Japan; 7 Present Address: Faculty of Agriculture, Tohoku University, Sendai, 980-8572 Japan

**Keywords:** Plant sciences, Plant biotechnology, Plant breeding, Plant cell biology, Plant physiology

## Abstract

Although plants have several advantages for foreign protein production, cultivation of transgenic plants in artificial plant growth facilities involves the use of a great amount of electricity for lightning and air conditioning, reducing cost-effectiveness. Protein production in plants grown in darkness can overcome this problem, but the amount of protein produced in the dark is unknown. In this study, the total amount of soluble protein produced in rice seedlings germinated and grown in light or darkness were examined at several time points after germination and under different temperature, nutritional, and seedling density conditions. Our results indicate that rice seedlings grown in darkness produce a comparable amount of total soluble protein to those grown in light. Furthermore, we found that the best conditions for protein production in dark-grown rice seedlings are large seeds germinated and grown for 10–12 days at 28 °C supplemented with Murashige and Skoog medium and 30 g/l sucrose in dense planting. Therefore, our results suggest that foreign proteins can be produced in rice seedlings in the dark, with a reduced electricity use and an increase in cost-effectiveness.

## Introduction

Plants have several advantages for the production of biological materials and chemicals over animals and microorganisms^1,2^. Plants can be cultivated cost-effectively, because they require only inorganic salts, water, light, moderate temperatures, and no expensive medium or nutrition for their growth. Plants have an extremely low probability of carrying pathogens that infect humans or livestock, including unidentified viruses^3^. Plants have been used to produce phytochemicals that support and enhance human health, and their extraction methods have continuously improved for their further use^4^. Plants can also be used for protein production. Proteins produced in plants can be stored for a long time at room temperature without refrigeration, especially if they are produced in seeds. Direct intake of proteins produced in edible plants or edible parts of plants has several applications, including oral vaccines.

However, plants generally have a lower ability to produce protein compared to animals and microorganisms. To overcome this disadvantage, numerous studies have been carried out to enhance the amount of foreign protein produced in plants, and various methods have been successfully developed to increase the protein yield^5^. One method is to add a transit signal sequence to the N-terminus of the protein of interest leading to protein accumulation in the chloroplasts, which show low proteinase activities compared to cytoplasm^6^. In some plant species such as tobacco (*Nicotiana tabacum*), direct transformation of chloroplasts is also possible^7^. Another method used in rice is to accumulate foreign proteins in the protein body of the endosperm. This method is especially useful for direct intake of proteins for oral vaccination^8^. The expression cassette has also been improved after the identification of several 5′-untranslated regions that are suitable for dicotyledonous and monocotyledonous plants and enhance translation efficiencies^9^. Nucleotide sequences around the translation initiation codon are also important for efficient translation, with those in *Arabidopsis* and rice being identified^10^. A terminator also affects the expression level of an introduced gene, and the terminator of heat shock protein gene was shown more efficient than the terminator of the nopaline synthase gene, which has been conventionally used^11,12^. These studies provided valuable information and tools to produce proteins in plants and clearly weakened the disadvantage of plants as bioreactors for protein production.

Transgenic plants carrying foreign genes to produce foreign proteins need to be grown in artificial plant growing facilities because of the legal regulations of genetically modified organisms, and in the case of therapeutic proteins, product and quality management. Therefore, cost-effectiveness, one of the main advantages of protein production in plants, is reduced due to plant cultivation under complete artificial conditions, which often uses a huge amount of electricity. Electricity is used for lightning, but most of the electric energy is finally converted into heat, which must be removed using air conditioning. Thus, a reduction in electricity consumption is essential for the cost-effective production of foreign proteins in plants. Plant cultivation in darkness under artificial conditions reduces electricity use and improves cost-effectiveness, and still complies with the transgenic regulations and the production management regulations. An etiolated sprout-producing factory previously used to produce bean sprout for consumption, would be a suitable location for protein production in plants. Furthermore, protein production in darkness is also important from a global environmental perspective^13,14^, because a huge amount of fossil fuels is still used for the generation of electricity.

Protein production under dark conditions involves bioconversion of already accumulated biomass to protein because de novo synthesis of biomass by photosynthesis cannot occur. Consequently, plant seeds that accumulate a certain amount of biomass can be used for protein production in darkness. One suitable candidate is rice, because it accumulates starch in the endosperm and there is already an established technique available for its genetic transformation allowing the introduction of a foreign gene^15^. However, it is not known how much protein is produced in rice under dark conditions. As the first step in effectively producing foreign proteins in darkness in rice, the amount of total soluble protein in rice seedlings that germinated and grew in darkness was examined. We also examined conditions that increase the protein amount in darkness.

## Results

### Quantification of total soluble protein produced in rice seedling under light and dark conditions

**Figure 1:**
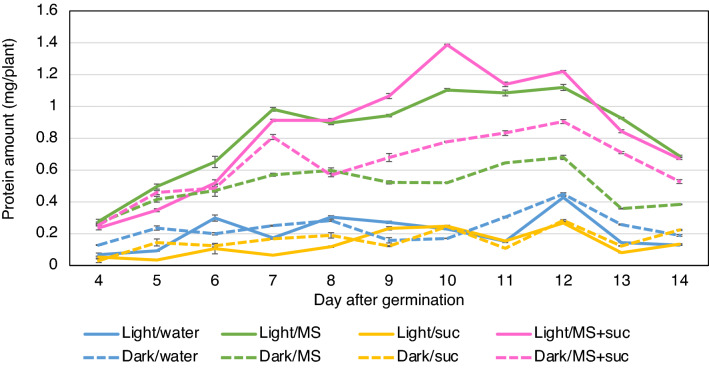
Protein production in rice seedlings grown under light and dark conditions with different nutritional supplements. The graph shows the amount of total soluble protein extracted from each seedling at 4–14 days after germination. Rice seeds were germinated in light or darkness and supplemented with Murashige and Skoog medium (MS) and/or 30 g/l sucrose (suc).

First, the amount of total soluble protein produced in rice (cultivar Nipponbare) seedlings germinated in light and darkness was examined. When seeds were germinated in water without supplementation, seedlings germinated in darkness produced a similar amount of total soluble protein as those germinated in light (Fig. 1). Both in light and darkness, the amount of total soluble protein reached a maximum at 12 days after germination (DAG), and each seedling in light produced 0.43 mg protein and that in darkness produced a similar protein amount (0.45 mg).

Next, the effects of supplementation with MS medium and sucrose on the amount of protein produced in each seedling were examined. Supplementation with MS medium alone increased the amount of protein in each seedling both in light and darkness, with co-supplementation with MS medium and 30 g/l sucrose further increasing this amount. When seedlings were grown in light and co-supplemented medium, each seedling produced 1.39 mg of protein at 10 DAG, which is 3.2-fold increase compared to that without supplementation at 12 DAG. When seedlings were grown in darkness, each seedling produced 0.90 mg of protein at 12 DAG, which is a 2.0-fold increase compared to that without supplementation at 12 DAG, but 35% less compared to that in light at 10 DAG. These results indicate that co-supplementation with MS medium and sucrose increases the maximum amount of total soluble protein obtained from seedlings grown under both light and dark conditions.

### Effects of specific groups of inorganic elements on protein production

**Figure 2:**
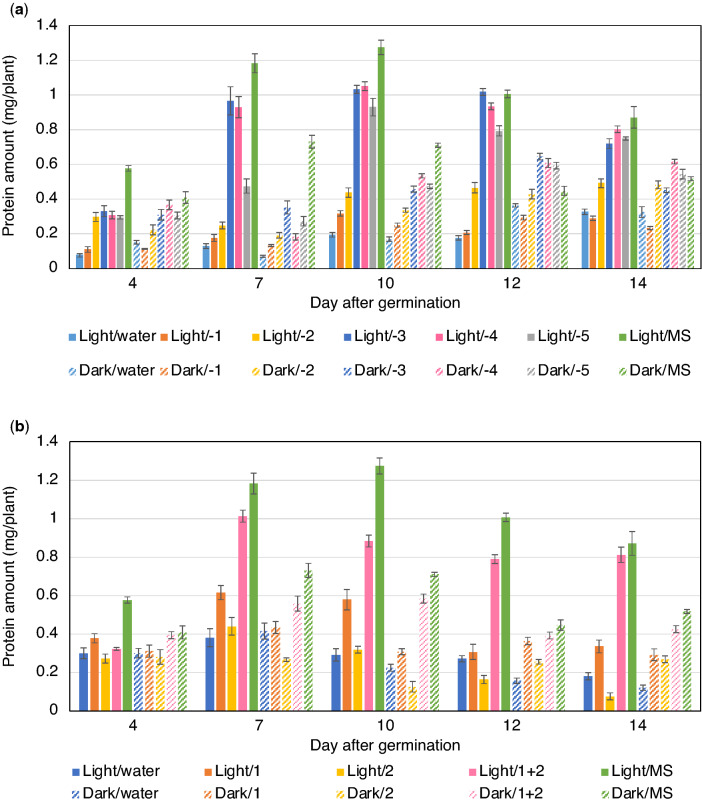
Protein production in rice seedlings supplemented with different combinations of the inorganic elements contained in the MS medium. (**a**) Total amount of soluble protein extracted from each seedling (n = 5) after supplementation with MS medium or MS medium variants lacking a specific group of inorganic components. Results of statistical analysis are shown in Supplementary Table S1. (**b**) Total amount of soluble protein extracted from each seedling (n = 5) after supplementation with group 1, group 2 or their combination. Results of statistical analysis are shown in Supplementary Table S2.

Since the MS medium contains various inorganic elements, we examined which specific components affected protein production. The inorganic elements of MS medium were divided into five groups (Table 1). To examine which group was more effective in increasing protein production, each of the five groups from the MS medium was removed and the seedlings were supplemented with the four remaining groups. The results showed that both in light and darkness removal of group 1 or group 2 greatly reduced the increase in the protein amount induced by supplementation with MS medium, whereas removal of groups 3, 4, or 5 had minor effects (Fig. 2a; Supplementary Table S1). Therefore, the effects of supplementation with group 1 alone, group 2 alone, and a combination of both was examined. The results showed that supplementation with either group 1 or group 2 showed no or little effect on the protein amount either in light or in darkness (Fig. 2b; Supplementary Table S2). Alternatively, co-supplementation with group 1 and group 2 was effective both in light and dark conditions, although the effect was lower than that of supplementation with complete MS medium (Fig. 2b; Supplementary Table S2).

**Table 1:** Inorganic elements of Murashige and Skoog medium.

Group	Element	Concentration (mg/l)
1	NH_4_NO_3_	1650
	KNO_3_	1900
2	MgSO_4_	181
	MnSO_4_	15
	ZnSO_4_	4.8
	CuSO_4_	0.016
3	CaCl_2_	332
	CoCl_2_	0.014
	KI	0.83
4	KH_2_PO_4_	170
	H_3_BO_3_	6.2
	Na_2_MoO_4_	0.21
5	Fe(III)-EDTA	43

**Figure 3:**
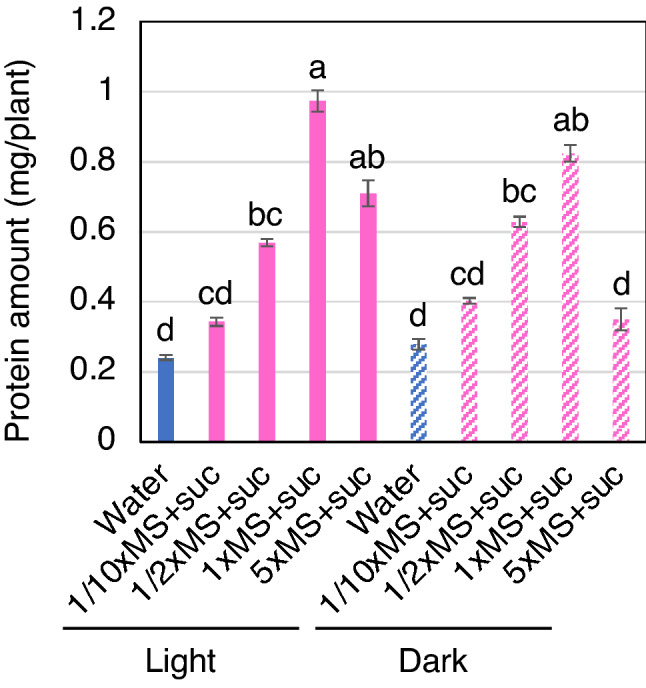
Protein production in rice seedlings using different concentrations of MS medium. The graph shows the total amount of soluble protein extracted from each seedling (n = 5) at 12 DAG in light or in darkness after supplementation with different concentrations of MS medium and 30 g/l sucrose. Different letters indicate significant difference by ANOVA at *P* < 0.05.

The effect of MS medium concentration on protein production was also examined. Under all conditions, 30 g/l sucrose was added to the MS medium, and total soluble protein was extracted at 12 DAG. When MS medium with 1/10 or half concentration of each element was used, the protein amount under light conditions was reduced to 35% and 58%, and in darkness 49% and 76%, respectively, compared to that produced with supplementation of original MS medium (Fig. 3). When fivefold strength MS medium was supplemented, the protein amount was 73% and 42% lower in light and darkness, respectively (Fig. 3). These results indicate that the most effective MS medium was the one containing the original nutrient contents.

**Figure 4:**
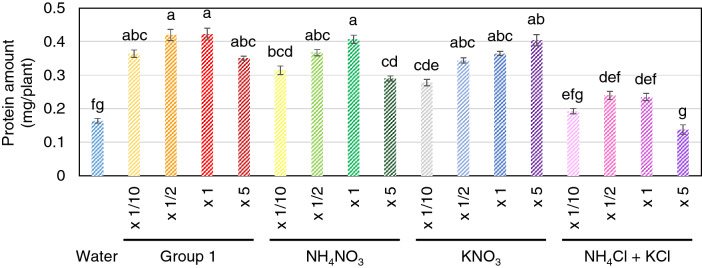
Protein production in rice seedlings grown in darkness and supplemented with different nitrogen type nutrients. The graph shows the total amount of soluble protein extracted from each seedling (n = 5) at 12 DAG in darkness after supplementation with different concentrations of group 1 nitrogen nutrients and 30 g/l sucrose. Different letters indicate significant difference by ANOVA at *P* < 0.05.

The effect of the concentration of group 1 in darkness was also examined. The results showed no significant differences in protein production in seedlings supplemented with concentrations ranging from 1/10 to 5-fold (Fig. 4). Because group 1 contained both NH_4_-type and NO_3_-type nitrogen nutrients, the effectiveness of both types was evaluated. The result showed that the NO_3_-type was more effective than the NH_4_-type in terms of protein production (Fig. 4).

### Effects of temperature on protein production

**Figure 5:**
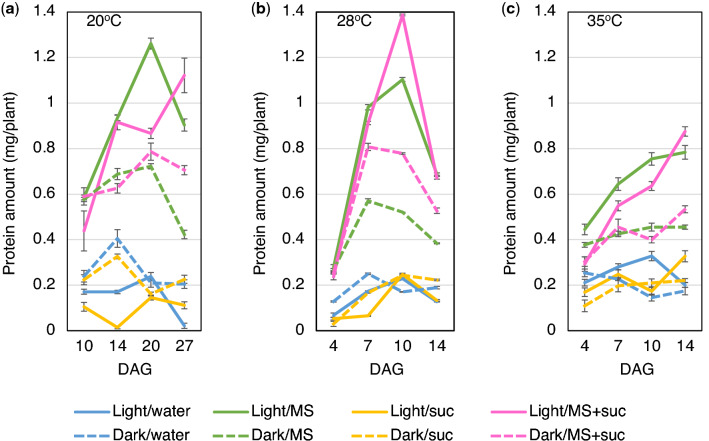
Protein production in rice seedlings grown at different temperatures. Graphs show the total amount of soluble protein extracted from each seedling (n = 5) grown at 20 °C (**A**), 28 °C (**B**), and 35 °C (**C**), and supplemented with MS medium and/or 30 g/l sucrose. DAG: day after germination.

The effects of three different temperatures (20 °C, 28 °C and 35 °C) on protein production in rice seedlings grown in either light or dark conditions and subjected to different nutritional supplements were examined. As expected, protein production at all temperatures was higher when seedlings were supplemented with MS medium alone or in combination with 30 g/l sucrose, and no significant differences between light and dark conditions were observed (Fig. 5). Interestingly, while temperature did not affect protein production when seedlings receive water or 30 g/l sucrose alone, it induced significant differences when seedlings were supplemented with MS medium. At 28 °C the maximum amount of protein was obtained at 10 DAG, which was 8 days faster than that at 20 °C (Fig. 5A,B). On the other hand, at 35 °C there was a 56% and 14% decrease in protein production in seedlings supplemented with MS and sucrose and MS alone, respectively, at 10 DAG in comparison to that at 28 °C. Altogether, these results indicate that 28 °C is a suitable temperature for protein production in rice seedlings.

### Effect of dense planting on protein production

**Figure 6:**
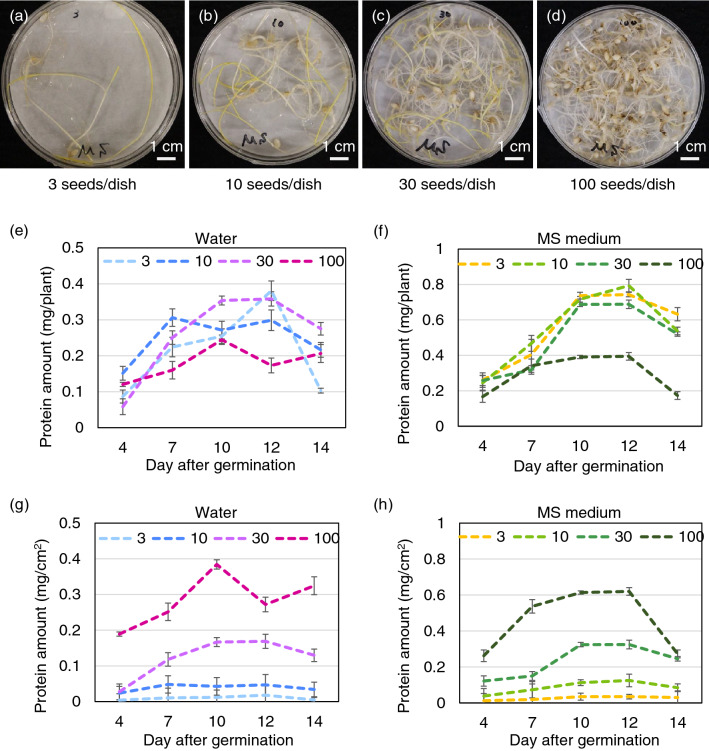
Effect of seedling density on the protein amount produced in rice seedlings. (**a**-**d**) Representative images of 3 (**a**), 10 (**b**), 30 (**c**), and 100 (**d**) rice seedlings at 14 DAG germinated and grown in a Petri dish in darkness and supplemented with MS medium. (**e**–**f**) Graphs show the total amount of soluble protein in each seedling (n = 5) germinated and grown in darkness with water (**e**) or supplemented with MS medium (**f**), and the protein amount per area in water (**g**) or MS medium (**h**).

The effects of seedling density on protein production were examined. Seeds (3–100) were germinated in a 9 cm diameter Petri dish and the total soluble protein was extracted at 4–14 DAG. Five well-grown seedlings were selected from the Petri dish at each density (Fig. 6a–d). When seeds were germinated in water at a density of 3–30 per dish, protein production was 0.30–0.35 mg of protein, whereas seedlings germinated at a 100-seed density produced only 0.24 mg at 10 DAG (Fig. 6e). Supplementation with MS medium increased the amount of protein in all density conditions; 0.69–0.79 mg protein was produced per seedling at a density of 3–30 per dish at 10 and 12 DAG, whereas only 0.39 mg of protein were produced at a 100-seed density (Fig. 6f).

The amount of protein produced per area was calculated based on the amount of protein produced in each seedling, and the number of seedlings per dish. The results showed that, in contrast to the amount of protein produced in each seedling, total protein produced per area was maximum at the density of 100 seeds per dish. When 100 seeds were germinated in a single petri dish, 0.38 mg protein/cm^2^ and 0.62 mg protein/cm^2^ were produced with water at 10 DAG and MS medium at 12 DAG, respectively (Fig. 6g, h). When 3–30 seeds were germinated in a single petri dish, a maximum of 0.02–0.17 mg protein/cm^2^ and 0.04–0.32 mg protein/cm^2^ were produced with water alone and MS medium, respectively (Fig. 6g, h).

Overall, these results indicate that although at high plant density the amount of protein produced in a single seedling is small, that condition increases protein production per area.

### Dependence of the protein amount on seed size

**Figure 7:**
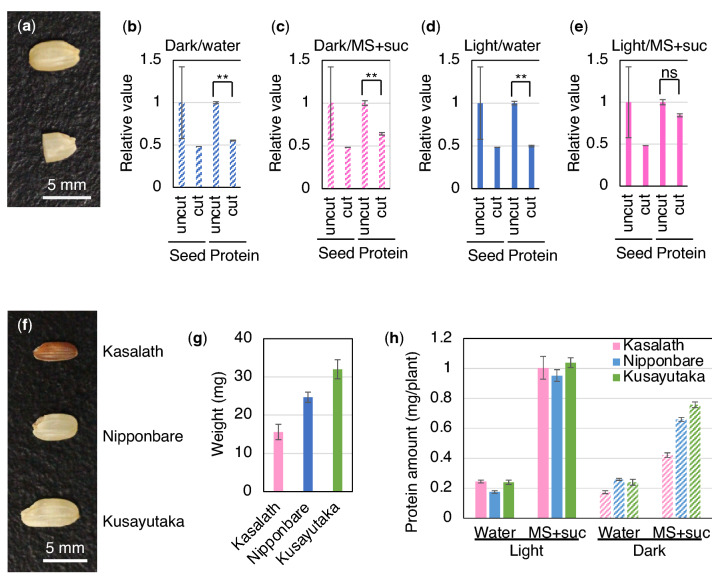
Effect of seed size on the protein amount produced in rice seedlings. (**a**) Representative picture of Nipponbare uncut and cut seeds. (**b**–**e**) Protein amount of Nipponbare seedlings germinated from uncut and cut seeds in darkness with water (**b**) and MS medium and 30 g/l sucrose (**c**), or in light with water (**d**) and MS medium and 30 g/l sucrose (**e**). Relative weight (n = 40) or protein amount (n = 5) is shown. The value of uncut seeds was set as 1. Left two bars show seed weight, and right two bars show the protein amount. **Significant difference by Student's *t* test (*P* < 0.01). ns: not significant (*P* > 0.05). (**f**) Representative picture of Kasalath, Nipponbare and Kusayutaka seeds. (**g**) Weight of Kasalath, Nipponbare and Kusayutaka seeds. The three cultivars showed significant difference by ANOVA at *P* < 0.05. (**h**) Total protein amount in Kasalath, Nipponbare and Kusayutaka seedlings. Seeds were germinated in light or in darkness with or without supplementation of MS medium and 30 g/l sucrose. Total soluble protein of each seedling (n = 5) was extracted at 11 DAG. No significant difference was observed between the three cultivars except between Kasalath and the other two cultivars in the condition with supplementation of MS medium and 30 g/l sucrose in darkness by ANOVA at *P* < 0.05.

Under dark conditions, photosynthesis cannot occur and proteins are produced from starch stored in the endosperm of rice seeds. This suggests that seed size affects may affect the amount of protein produced in seedlings germinated and grown in darkness. To examine this possibility, seeds were cut in half, and the half-sized seeds containing the embryo were subjected to protein production in darkness (Fig. 7a). The amount of protein produced in each half-sized seedling was 55% and 64% lower than that of the full-size seeds when they were germinated with water and supplemented with MS medium and 30 g/l sucrose, respectively (Fig. 7b, c). Although no significant reduction was observed with supplementation, the protein amount in light-grown seedlings from the half-sized seeds was also reduced (Fig. 7d, e). These results indicate that seed size affects protein production in each seedling in darkness, but the effect is less prominent in the absence of nutritional supplementation.

Next, the amount of protein produced by seedlings of the rice cultivars Kasalath and Kusayutaka was compared to that of Nipponbare, because the seed size of these cultivars is different. Kasalath produces small seeds, Nipponbare medium, and Kusayutaka large seeds (Fig. 7f, g). Seeds of these cultivars were germinated and grown in darkness in water or with supplementation with MS medium and 30 g/l sucrose. A clear correlation between seed size and the amount of protein produced in each seedling was observed only with nutritional supplementation (Fig. 7h); suggesting that seed size affects protein production under certain conditions.

## Discussion

In the present study, the amount of total soluble protein in each seedling germinated and grown in light or in darkness was examined. Seedlings germinated and grown in water without nutritional supplementation produced a similar amount of total soluble protein. Supplementation with MS medium increased the protein amount both in light and in darkness, and in light, when 30 g/l sucrose was also added, the protein amount reached threefold more compared to that without supplementation. In darkness, the protein amount with supplementation with MS medium and 30 g/l sucrose was twofold higher compared to that without supplementation and was approximately 65% of that in light. Dense planting would be effective for the efficient use of plant growing facilities, as it increases the amount of protein produced per area. These results provide valuable information for the future production of useful proteins under cost-effective dark conditions.

Using large amount of antibiotics in human medical care and livestock production has resulted in the generation and widespread of multidrug-resistant pathogens, which are becoming a great threat to human health^16–18^. Antimicrobial proteins and peptides are good alternatives to antibiotics^19–21^, and still show antimicrobial activity against antibiotic-resistant pathogens^22,23^. One well-known antimicrobial protein is lysostaphin from *Staphylococcus simulans*, an enzyme that degrades the pentaglycine bridge in the cell wall of *Staphylococcus areus*^24^. In dairy cattle infected with *S. aureus*, an injection of lysostaphin through the teat canal cured the animals, and expression of lysostaphin in transgenic cows prevents the mastitis caused by *S. aureus*^25,26^. Despite the effectiveness of antimicrobial proteins and peptides to prevent pathogen infection and to cure infected animals, their practical application, especially for livestock, is limited due to the high cost of their production^27^. Therefore, the development of methods to produce proteins and peptides at very low cost is desired, and protein production in dark-grown plants would help to accomplish this and support the practical use of antimicrobial proteins and peptides.

Rice has several advantages in terms of protein production in plants^28^. Genetic engineering methods have also been developed for this purpose. An efficient *Agrobacterium*-mediated transformation is available^15^, and gene knockout by homologous recombination as well as genome editing, both in nucleus and mitochondrion, are possible^29,30^. Rice is a staple food for humans and is used for feeding livestock, which guarantees safety based on eating experience by reducing unexpected contamination of purified proteins with toxic compounds^31^. This also makes oral intake of proteins feasible^8,32^. For protein production in darkness, seed size is an important factor, and rice grains are filled with starch that can be converted into protein^33,34^. These characteristics render rice a suitable plant for protein production in the dark.

Light is known to regulate gene expression in plants^35^. For example, the expression of genes encoding photosynthesis-related proteins is induced in light and reduced in darkness^36,37^. However, genes upregulated in the dark have also been reported^38^. The most abundant protein in plants grown under light conditions is the Rubisco large subunit, which along with other photosynthetic proteins occupy approximately half of the total soluble protein^39^. This means that under light conditions, half of the protein-producing ability and resources of the plants are used for the production of photosynthetic proteins, and therefore the remaining half produces foreign proteins. If plants germinate and grow under dark conditions, the production of photosynthetic proteins is repressed^36,37^, and higher protein production ability and resources are applied for the production of useful proteins. Thus, it is suggested that, under dark conditions, foreign protein production may increase compared to that under the light, although this possibility needs to be examined.

Production of protein in darkness in plants requires the bioconversion of accumulated photosynthetic products, such as starch and lipids, to protein. In the case of seedlings germinated in darkness, starch or lipids in the seeds are converted to protein. This suggests that larger seeds with greater accumulation of photosynthetic products can produce more protein in the dark. In agreement with this, the results of the current study showed that rice seeds cut in half produced less protein than full-size seeds at 12 DAG. However, Kusayutaka, which sets large seeds^40^, produced a similar amount of protein to Kasalath and Nipponbare, which set small and medium size seeds, respectively, in the absence of nutritional supplementation. This suggests the presence of factors that determine protein amount in dark-grown seedlings other than seed size, or a mechanism to compensate for the protein amount caused by the difference in seed size. The availability of inorganic elements is one of the candidates, because supplementation with MS medium showed a positive correlation between seed size and the protein amount. Further analysis of the factors that regulate protein levels is necessary to increase protein production.

In conclusion, we found that the best conditions for protein production in dark-grown rice seedlings are large seeds germinated and grown for 10–12 days at 28 °C supplemented with MS medium and 30 g/l sucrose in dense planting. Among these conditions, supplementation with MS medium had the greatest effect on the protein amount. Our results suggest that foreign protein production in rice is feasible in darkness, which reduces the cost of electricity for lightning and air conditioning. Future studies are required to evaluate foreign protein production in dark-grown rice seedlings. First, it is important to examine whether the same conditions can be applied for the efficient production of different proteins, or if each protein has to be produced under specific conditions. Second, suitable subcellular localization for high protein accumulation needs evaluation for each protein, because it varies between different proteins^32,41^. These studies will provide additional necessary information for the production of foreign proteins in darkness with reduced use of electricity, and thus, at lower cost.

## Material and methods

All the experiments were performed in accordance with relevant guidelines and regulations.

### Plant materials

Rice (*Oryza sativa* L.) cultivar Nipponbare was used in all experiments. Rice cultivars Kasalath and Kusayutaka were used only for the seed-size experiments. We obtained all necessary permissions to use these cultivars.

### Seed germination and growth

Seeds were dehusked, surface-sterilized, placed on a filter paper with 5 ml of water in a 9 cm plastic Petri dish and incubated at 28 °C for different number (4–14) of days in continuous light or dark conditions in a growth chamber. To evaluate the effect of temperature, seeds were incubated at 20, 28, or 35 °C. Ten seeds were placed in a single Petri dish in all cases, except in the case of the density experiments, in which 3, 10, 30 or 100 seeds were used. Additionally, nutritional supplementation with MS medium (pH 5.8) and/or 30 g/l sucrose was used in nutrition-supplemented experiments. In the experiment of the effect of seed size, seeds were cut in half with a surgical knife.

### Protein extraction and concentration measurement

Five well-grown seedlings were selected from each Petri dish and subjected to protein extraction. First, the remaining seed portion was removed from the seedlings. Then, seedlings at 6 DAG or less were ground completely with mortar and pestle in 0.5 ml of Tris-buffered saline (TBS). Seedlings at 7 DAG or more were frozen in liquid nitrogen, ground into powder, and mixed thoroughly after adding 1 ml of TBS. After centrifugation at 14,000 rpm for 5 min at 4 °C, the supernatant was collected and used as the extract of total soluble protein. Protein concentration was measured with the Bradford protein assay (Bio-Rad) using bovine serum albumin as a standard. The amount of total soluble protein in a seedling was calculated based on the protein concentration and the volume of the protein extracts, and the average of five seedlings is shown for each experiment. Analysis of variance (ANOVA) or Student's t-test was carried out for a statistic analysis.

## Supplementary Information

Supplementary Information 1.

Supplementary Information 2.

## Data Availability

All data generated or analyzed during this study are included in this published article and its supplementary information files.
