# Supplementary material for: Evaluation of protein production in rice seedlings under dark conditions
Source: Sci Rep. 2022 May 11;12:7759. doi: 10.1038/s41598-022-11672-0 (PMC9095683; doi:10.1038/s41598-022-11672-0)
Supplement: Supplementary file 2 — Supplementary Information 2. [file 41598_2022_11672_MOESM2_ESM.pdf]

Title: Evaluation of protein production in rice seedlings under dark conditions

Authors: Akiko Watanabe, Yoshino Hatanaka, Yukino Takeshima, Karin Sasaki, Noa Takahashi, Yukihiro Ito

Table S1. Statistic analysis of data shown in Figure 2A

		Light/water	Light/-1	Light/-2	Light/-3	Light/-4	Light/-5	Light/MS	Dark/water	Dark/-1	Dark/-2	Dark/-3	Dark/-4	Dark/-5	Dark/MS
	Light/water	-													
	Light/-1	ns	-												
	Light/-2	s	s	-											
	Light/-3	s	s	ns	-										
	Light/-4	s	s	ns	ns	-									
	Light/-5	s	s	ns	ns	ns	-								
4 DAG	Light/MS	s	s	s	s	s	s	-							
	Dark/water	ns	ns	ns	s	ns	ns	s	-						
	Dark/-1	ns	ns	s	s	s	s	s	ns	-					
	Dark/-2	ns	ns	ns	ns	ns	ns	s	ns	ns	-				
	Dark/-3	s	s	ns	ns	ns	ns	s	s	s	ns	-			
	Dark/-4	s	s	ns	ns	ns	ns	s	s	s	ns	ns	-		
	Dark/-5	s	s	ns	ns	ns	ns	s	s	s	ns	ns	s	-	
	Dark/MS	s	s	ns	ns	ns	ns	s	s	s	s	ns	ns	ns	-
	Light/water	-													
	Light/-1	ns	-												
	Light/-2	ns	ns	-											
	Light/-3	s	s	s	-										
	Light/-4	s	s	s	ns	-									
	Light/-5	ns	ns	ns	s	ns	-								
7 DAG	Light/MS	s	s	s	ns	ns	s	-							
	Dark/water	ns	ns	ns	s	s	ns	s	-						
	Dark/-1	ns	ns	ns	s	s	ns	s	ns	-					
	Dark/-2	ns	ns	ns	s	s	ns	s	ns	ns	-				
	Dark/-3	ns	ns	ns	s	s	ns	s	ns	ns	ns	-			
	Dark/-4	ns	ns	ns	s	s	ns	s	ns	ns	ns	ns	-		
	Dark/-5	ns	ns	ns	s	s	ns	s	ns	ns	ns	ns	ns	-	
	Dark/MS	s	s	s	ns	ns	ns	ns	s	s	s	ns	s	s	-
	Light/water	-													
	Light/-1	ns	-												
	Light/-2	ns	ns	-											
	Light/-3	s	s	s	-										
	Light/-4	s	s	s	ns	-									
	Light/-5	s	s	s	ns	ns	-								
10 DAG	Light/MS	s	s	s	ns	ns	s	-							
	Dark/water	ns	ns	ns	s	s	s	s	-						
	Dark/-1	ns	ns	ns	s	s	s	s	ns	-					
	Dark/-2	ns	ns	ns	s	s	s	s	ns	ns	-				
	Dark/-3	ns	ns	ns	s	s	s	s	s	ns	ns	-			
	Dark/-4	s	ns	ns	s	s	s	s	s	s	ns	ns	-		
	Dark/-5	s	ns	ns	s	s	s	s	s	ns	ns	ns	ns	-	
	Dark/MS	s	s	ns	s	s	ns	s	s	s	s	ns	ns	ns	-
	Light/water	-													
	Light/-1	ns	-												
	Light/-2	s	s	-											
	Light/-3	s	s	s	-										
	Light/-4	s	s	s	ns	-									
	Light/-5	s	s	s	ns	ns	-								
12 DAG	Light/MS	s	s	s	ns	ns	ns	-							
	Dark/water	ns	ns	ns	s	s	s	s	-						
	Dark/-1	ns	ns	ns	s	s	s	s	ns	-					
	Dark/-2	s	ns	ns	s	s	s	s	ns	ns	-				
	Dark/-3	s	s	ns	s	s	ns	s	s	s	ns	-			
	Dark/-4	s	s	ns	s	s	ns	s	ns	s	ns	ns	-		
	Dark/-5	s	s	ns	s	s	ns	s	ns	s	ns	ns	ns	-	
	Dark/MS	s	ns	ns	s	s	s	s	ns	ns	ns	ns	ns	ns	-
	Light/water	-													
	Light/-1	ns	-												
	Light/-2	ns	ns	-											
	Light/-3	s	s	ns	-										
	Light/-4	s	s	s	ns	-									
	Light/-5	s	s	ns	ns	ns	-								
14 DAG	Light/MS	s	s	s	ns	ns	ns	-							
	Dark/water	ns	ns	ns	s	s	s	s	-						
	Dark/-1	ns	ns	ns	s	s	s	s	ns	-					
	Dark/-2	ns	ns	ns	ns	s	ns	s	ns	ns	-				
	Dark/-3	ns	ns	ns	ns	s	ns	s	ns	ns	ns	-			
	Dark/-4	ns	s	ns	ns	ns	ns	ns	ns	s	ns	ns	-		
	Dark/-5	ns	ns	ns	ns	ns	ns	s	ns	s	ns	ns	ns	-	
	Dark/MS	ns	ns	ns	ns	ns	ns	s	ns	ns	ns	ns	ns	ns	-

s: significant difference by ANOVA ($P < 0.05$), ns: not significant by ANOVA ($P > 0.05$).

Table S2. Statistic analysis of data shown in Figure 2B

		Light/water	Light/1	Light/2	Light/1+2	Light/MS	Dark/water	Dark/1	Dark/2	Dark/1+2	Dark/MS
4 DAG	Light/water	-									
	Light/1	ns	-								
	Light/2	ns	ns	-							
	Light/1+2	ns	ns	ns	-						
	Light/MS	s	s	s	s	-					
	Dark/water	ns	ns	ns	ns	s	-				
	Dark/1	ns	ns	ns	ns	s	ns	-			
	Dark/2	ns	ns	ns	ns	s	ns	ns	-		
	Dark/1+2	ns	ns	ns	ns	ns	ns	ns	ns	-	
	Dark/MS	ns	ns	ns	ns	ns	ns	ns	ns	ns	-
7 DAG	Light/water	-									
	Light/1	ns	-								
	Light/2	ns	ns	-							
	Light/1+2	s	s	s	-						
	Light/MS	s	s	s	ns	-					
	Dark/water	ns	ns	ns	s	s	-				
	Dark/1	ns	ns	ns	s	s	ns	-			
	Dark/2	ns	s	ns	s	s	ns	ns	-		
	Dark/1+2	ns	ns	ns	s	s	ns	ns	s	-	
	Dark/MS	s	ns	ns	ns	s	s	ns	s	ns	-
10 DAG	Light/water	-									
	Light/1	ns	-								
	Light/2	ns	ns	-							
	Light/1+2	s	ns	s	-						
	Light/MS	s	s	s	s	-					
	Dark/water	ns	s	ns	s	s	-				
	Dark/1	ns	s	ns	s	s	ns	-			
	Dark/2	ns	s	ns	s	s	ns	ns	-		
	Dark/1+2	ns	ns	ns	ns	s	s	s	s	-	
	Dark/MS	s	ns	s	ns	s	s	s	s	ns	-
12 DAG	Light/water	-									
	Light/1	ns	-								
	Light/2	ns	ns	-							
	Light/1+2	s	s	s	-						
	Light/MS	s	s	s	ns	-					
	Dark/water	ns	ns	ns	s	s	-				
	Dark/1	ns	ns	ns	s	s	ns	-			
	Dark/2	ns	ns	ns	s	s	ns	ns	-		
	Dark/1+2	ns	ns	ns	s	s	ns	ns	ns	-	
	Dark/MS	ns	ns	s	s	s	s	ns	ns	ns	-
14 DAG	Light/water	-									
	Light/1	ns	-								
	Light/2	ns	ns	-							
	Light/1+2	s	s	s	-						
	Light/MS	s	s	s	ns	-					
	Dark/water	ns	ns	ns	s	s	-				
	Dark/1	ns	ns	ns	s	s	ns	-			
	Dark/2	ns	ns	ns	s	s	ns	ns	-		
	Dark/1+2	ns	ns	s	s	s	ns	ns	ns	-	
	Dark/MS	s	ns	s	ns	s	s	ns	ns	ns	-

s: significant difference by ANOVA ($P < 0.05$), ns: not significant by ANOVA ($P > 0.05$).
